# Automated Processing of Imaging Data through Multi-tiered Classification of Biological Structures Illustrated Using *Caenorhabditis elegans*

**DOI:** 10.1371/journal.pcbi.1004194

**Published:** 2015-04-24

**Authors:** Mei Zhan, Matthew M. Crane, Eugeni V. Entchev, Antonio Caballero, Diana Andrea Fernandes de Abreu, QueeLim Ch’ng, Hang Lu

**Affiliations:** 1 Interdisciplinary Program in Bioengineering, Georgia Institute of Technology, Atlanta, Georgia, United States of America; 2 Wallace H. Coulter Department of Biomedical Engineering, Georgia Institute of Technology, Atlanta, Georgia, United States of America; 3 MRC Centre for Developmental Neurobiology, Kings College London, London, United Kingdom; 4 School of Chemical & Biomolecular Engineering, Georgia Institute of Technology, Atlanta, Georgia, United States of America; University of Illinois at Urbana-Champaign, UNITED STATES

## Abstract

Quantitative imaging has become a vital technique in biological discovery and clinical diagnostics; a plethora of tools have recently been developed to enable new and accelerated forms of biological investigation. Increasingly, the capacity for high-throughput experimentation provided by new imaging modalities, contrast techniques, microscopy tools, microfluidics and computer controlled systems shifts the experimental bottleneck from the level of physical manipulation and raw data collection to automated recognition and data processing. Yet, despite their broad importance, image analysis solutions to address these needs have been narrowly tailored. Here, we present a generalizable formulation for autonomous identification of specific biological structures that is applicable for many problems. The process flow architecture we present here utilizes standard image processing techniques and the multi-tiered application of classification models such as support vector machines (SVM). These low-level functions are readily available in a large array of image processing software packages and programming languages. Our framework is thus both easy to implement at the modular level and provides specific high-level architecture to guide the solution of more complicated image-processing problems. We demonstrate the utility of the classification routine by developing two specific classifiers as a toolset for automation and cell identification in the model organism *Caenorhabditis elegans*. To serve a common need for automated high-resolution imaging and behavior applications in the *C*. *elegans* research community, we contribute a ready-to-use classifier for the identification of the head of the animal under bright field imaging. Furthermore, we extend our framework to address the pervasive problem of cell-specific identification under fluorescent imaging, which is critical for biological investigation in multicellular organisms or tissues. Using these examples as a guide, we envision the broad utility of the framework for diverse problems across different length scales and imaging methods.

## Author Summary

New technologies have increased the size and content-richness of biological imaging datasets. As a result, automated image processing is increasingly necessary to extract relevant data in an objective, consistent and time-efficient manner. While image processing tools have been developed for general problems that affect large communities of biologists, the diversity of biological research questions and experimental techniques have left many problems unaddressed. Moreover, there is no clear way in which non-computer scientists can immediately apply a large body of computer vision and image processing techniques to address their specific problems or adapt existing tools to their needs. Here, we address this need by demonstrating an adaptable framework for image processing that is capable of accommodating a large range of biological problems with both high accuracy and computational efficiency. Moreover, we demonstrate the utilization of this framework for disparate problems by solving two specific image processing challenges in the model organism *Caenorhabditis elegans*. In addition to contributions to the *C*. *elegans* community, the solutions developed here provide both useful concepts and adaptable image-processing modules for other biological problems.

This is a *PLOS Computational Biology* Methods paper

## Introduction

**Fig 1:**
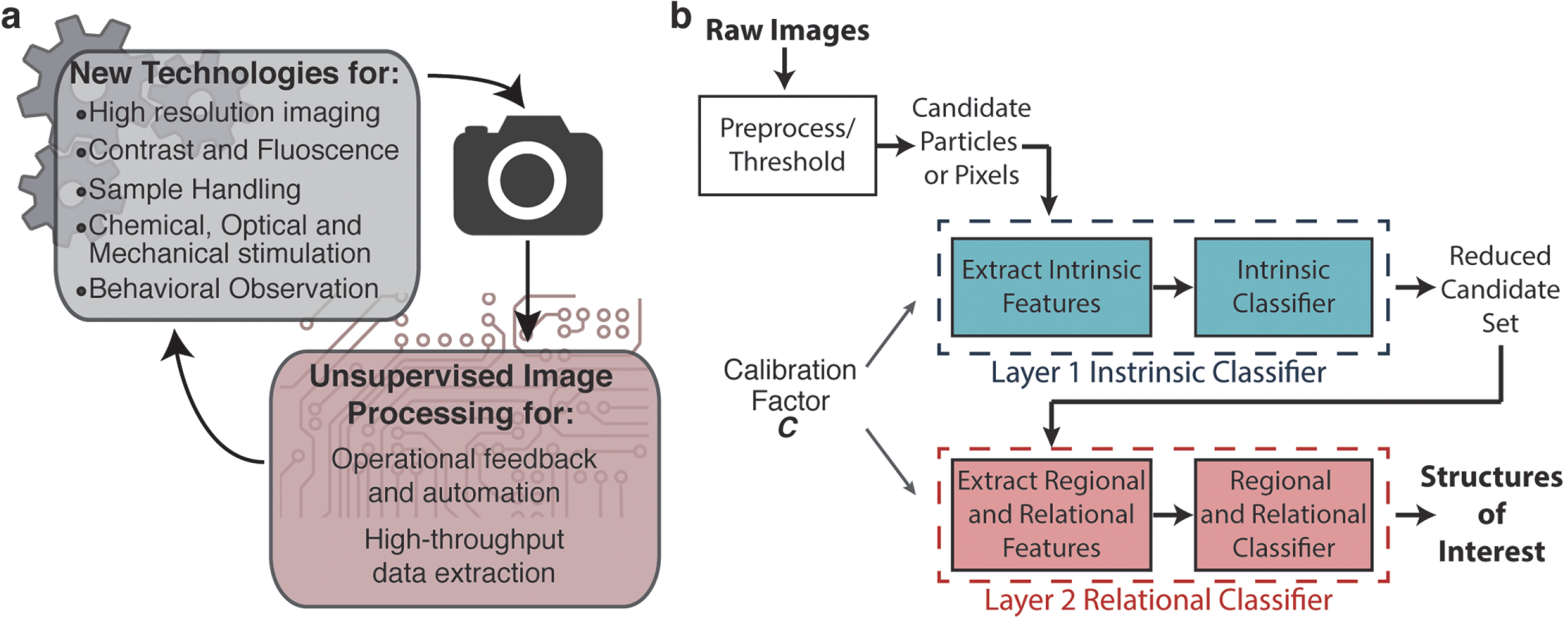
Overview of biological structure detection using multi-tiered classification. a) Unsupervised image processing techniques are often necessary to harness the power of emerging imaging and experimental technologies. b) An overview of the proposed generalizable two layer classification architecture for the autonomous identification of specific biological structures. Intrinsic, computationally simple features and relational or computationally expensive features are partitioned into two layers to accommodate both structural complexity and efficiency.

Diverse imaging techniques exist to provide functional and structural information about biological specimens in clinical and experimental settings. On the clinical side, new and augmented imaging modalities and contrast techniques have increased the types of information that can be garnered from biological samples [1]. Similarly, many tools have recently been developed to enable new and accelerated forms of biological experimentation in both single cells and multicellular model organisms [2–10]. Increasingly, the capacity for high-throughput experimentation provided by new optical tools, microfluidics and computer controlled systems has eased the experimental bottleneck at the level of physical manipulation and raw data collection. Still, the power of many of these toolsets lies in facilitating the automation of experimental processes. The ability to perform real-time information extraction from images during the course of an experiment is therefore a crucial computational step to harnessing the potential of many of these physical systems (Fig 1A). Even when off-line data analysis is sufficient, the capability of these systems to generate large, high-content datasets places a large burden on the speed of the downstream analysis.

Automated image processing and the use of supervised learning techniques have the potential for bridging this gap between raw data availability and the limitations of manual analysis in terms of speed, objectivity and sensitivity to subtle changes [11]. In this area, many computer vision techniques, including some general object detection strategies, have been developed to address the detection and recognition of faces, vehicles, animals and household objects from standard camera images [12–17]. While this body of literature solves complex recognition problems within the domain of everyday objects and images, it is not clear how or whether they are generalizable to the imaging modalities and object detection problems that arise in biological image processing. While these techniques have garnered some important but limited adoption in biological applications[18–28], there is not a systematic methodology by which these computational approaches can be applied to solving common problems in mining biological images [29]. Thus, the development or adaptation of these tools for specific problems has thus far been relatively opaque to many potential end-users and require a high degree of expertise and intuition.

At the same time, there is a diverse array of specific object recognition problems that arise in biology. Specifically, extraction of meaningful information from biological images usually involves the identification of particular structures and calculation of their metrics, rather than the usage of global image metrics. Depending on the specimen and the experimental platform, this may range in scale from molecular or sub-cellular structure to individual cells or tissue structures within a heterogeneous specimen, or entire organisms. While toolsets have already been developed to address some common needs in biology [19–22, 24, 25, 30–32] and while powerful algorithmic tools exist for pattern and feature discrimination and decision-making [33–35], there are still many unaddressed needs in biological image processing.

Here, we present a general scheme for the detection of specific biological structures applicable as a basis for solving a broad set of problems while using non-specific image processing modules. As opposed to finished, ready-to-use toolsets, which address a limited problem definition by design, the workflow we propose has the power to simultaneously address the need for accuracy, problem-specificity, and generalizability; end-users have the opportunity to choose platforms and customize as needed. We demonstrate the power of this approach for solving disparate biological image processing problems by developing two widely relevant toolsets for the multicellular model organism, *Caenorhabditis elegans*. To address the problems of extracting region-, tissue- and cell-specific information within a multicellular context, we developed an image processing algorithm to distinguish the head of the worm under bright-field imaging and a set of tools for specific cell identification under fluorescence imaging. These developments demonstrate the flexibility of our framework to accommodate different imaging modalities and disparate biological structures. The resulting toolsets contribute directing to addressing two fundamental needs for automated studies in the worm and contribute specific concepts and modules that may be applied to a broader range of biological problems.

## Results

Our framework is a two-tiered classification scheme to identify specific biological structures within an image (Fig 1B). To identify biological structures of interest, images are first pre-processed to condition the data and generate candidates for the structure of interest. In general, candidates can either be individual pixels or discrete segmented regions generated via a thresholding algorithm applied during pre-processing. To accommodate different image acquisition setups and acquisition parameters, we propose the use of an image calibration factor, *C*, in preprocessing and in all subsequent feature calculation steps. This calibration factor characterizes the relationship between the digitized and real-world length scales for a specific experimental setup and can be used to normalize feature and parameter scaling in all image processing steps (Materials and Methods, S1 Table).

Subsequently we apply a two-layer classification scheme to identify whether the candidates generated are features of interest. The candidate particles are quantitatively described by two distinct sets of descriptive features. These features may be derived from intuitive metrics designed to mimic human recognition or abstractions that capture additional information [33, 36]; they are mathematical descriptors that help delineate the structures of interest from other candidates and will form the basis for classification. Separation of features into two distinct layers of classification in our proposed scheme serves three purposes. First, it permits conceptual separation of intrinsic and extrinsic or relational properties of a biological structure. Second, it permits the inclusion of higher level descriptions of the relationships between structures identified from the first layer of classification. Finally, it allows computationally expensive features to only be associated with the second layer, which reduces the number of times these features must be calculated as low probability candidates have already been removed. Accordingly, the first layer of classification uses computationally inexpensive, intrinsic features of the candidates to generate a smaller set of candidates. The second layer addresses additional complexity, and uses computationally more expensive features or extrinsic features describing the relationship between candidates, but only on a smaller number of candidates. This two-tier scheme allows significant reduction in computational time. At each layer of classification, a trained classifier is used to make a decision about the candidate’s identity based on the features calculated. In this work, we chose to use support vector machines for all classification steps because of its insensitivity to specific conditioning of feature sets and therefore being more robust [34, 37]. We note that when constraints of the feature sets are well known, other models including Bayesian discriminators and heuristic thresholds can also be used. In general, the workflow architecture presented in Fig 1B permits the identification of generic biological structures and balances the capability for complexity with computational speed. We describe here two distinct applications using this two-tier classification methodology.

### Bright-Field Head Identification

**Fig 2:**
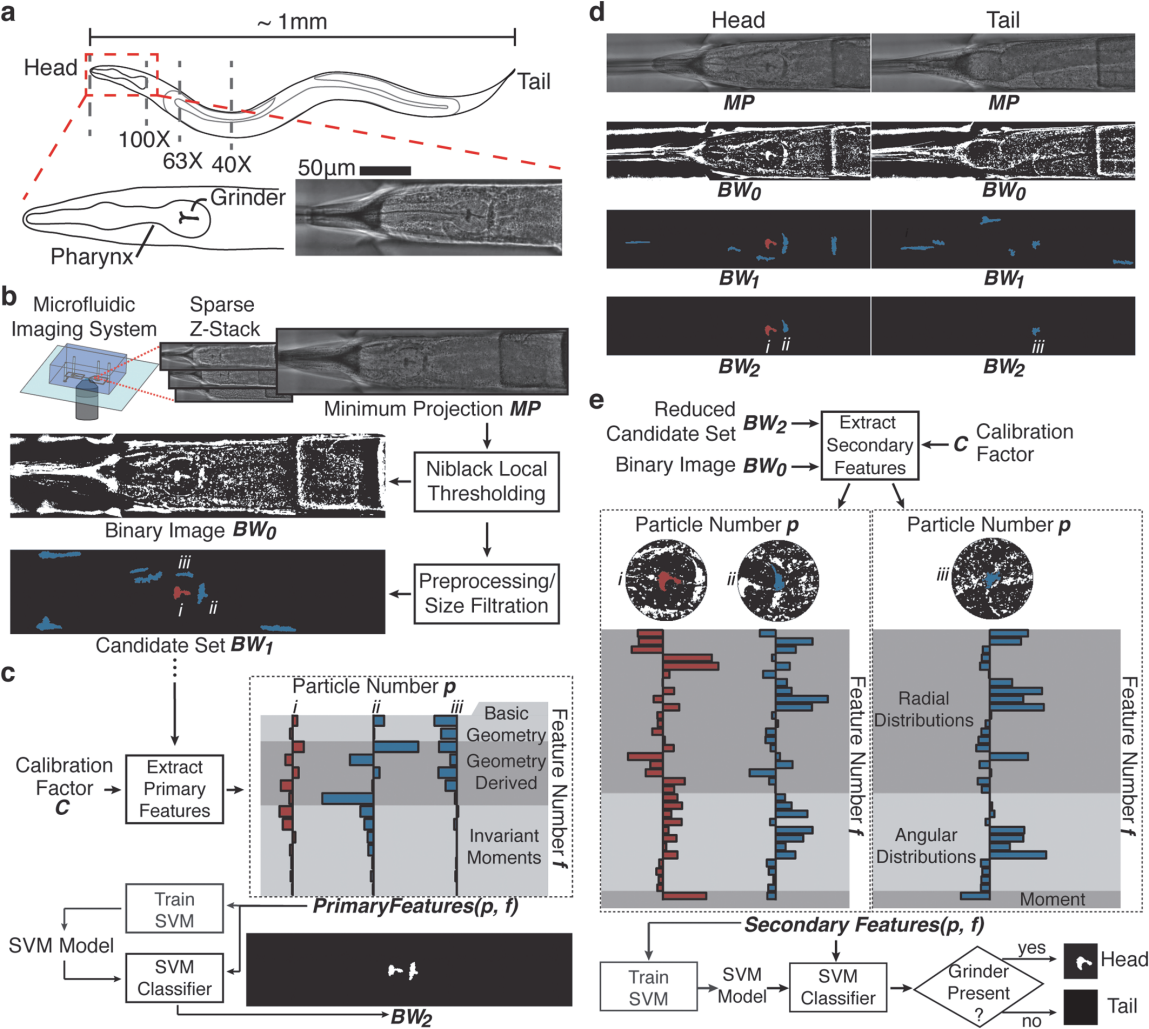
Preprocessing and feature selection for head versus tail discrimination in *C*. *elegans*. a) The limited field of view of high resolution imaging systems creates a need for spatial positioning along the anterior-posterior axis of the worm. As a landmark for orienting the A-P axis, the head of the worm is distinguished by the presence of the pharynx and a grinder structure (inset below). b) Preprocessing for bright field structural detection consists of minimum intensity projection of a sparse z-stack (*MP*) followed by Niblack local thresholding (*BW*_0_) and preliminary filtration of segmented particles to generate candidates for subsequent classification (*BW*_1_). c) In layer 1 of classification, computationally inexpensive, intrinsic properties of the candidates (*BW*_1_) are calculated for SVM classification and reduction of the candidate pool (*BW*_2_). d) Two example image processing sequences showing that while the shape-intrinsic features used in layer 1 of classification significantly reduces the candidate pool, it is insufficient for robust, specific identification of the grinder particle. e) From the reduced candidate pool, layer 2 of classification utilizes regional properties of the remaining candidates to distinguish the grinder from other structural and textural elements of the worm body with high specificity, making identification of the head possible on the basis of the presence of the grinder particle.

Due to its relatively large size, only a limited portion of the adult worm body can be captured within the field of view under high-resolution imaging; yet it is necessary to target specific regions along the anterior-posterior axis of the worm to capture or apply experimental perturbations to specific cells or tissues of interest (Fig 2A). Thus, image processing for orientation along the anterior-posterior axis of the worm is crucial to enabling the full potential of many of the toolsets for high-resolution imaging and physical, chemical and optical manipulation of the worm. To address this need, many *ad hoc* tactics such as the presence of fluorescent markers [5, 24, 38, 39] or the assumption of forward locomotion in freely moving worms [22, 25, 32, 40–43] are often used delineate between the head and tail and orient the anterior-posterior axis. However, reliance on exogenously introduced fluorescent markers can necessitate time-consuming treatment of the worms under study and can spatially interfere with other fluorescent readouts of interest. While the assumption of forward locomotion does not require additional treatments, it is only useful in experimental contexts where worms are freely mobile. Therefore, these tactics lack general applicability to many high resolution imaging experiments, where worms may lack appropriate fluorescent markers or are physically restrained or chemically immobilized. Additionally, not relying on fluorescent markers avoids unnecessary photobleaching of the sample before data acquisition and affords robustness against age and condition-specific autofluorescence in the worm body [44].

In order to approach this problem with minimal reliance on specific experimental conditions, we note several consistent morphological differences between the head and the tail of the worm that are observable in bright-field imaging. Bright-field is a commonly available imaging modality and often used for location and positioning of specimens prior to fluorescent imaging. While the shape of the head and the tail differs somewhat, these differences are difficult to detect due to low contrast and may be physically obscured by some experimental platforms [38]. Instead, the head of the worm is more clearly distinguished by the presence of the pharynx, which has a stereotypical morphology that includes a biological structure for masticating food called the grinder [45]. As shown in Fig 2A, the grinder is a dark, uniquely shaped, high-contrast structure under bright field imaging. The grinder can also be easily resolved by most digital cameras at imaging magnifications above 20X and maintains its shape and integrity for several days of early adulthood [46, 47]. This stereotypical feature of the head, which is relatively consistent in the worm post-developmentally, can thus serve as the target biological structure for our two-layer classification scheme.

To construct and validate our classification scheme, bright-field images of the worm head and tail were collected using a custom microfluidic device (Materials and Exp. Methods), although similar images on agar pad would also suffice (S1 Fig). Following our architecture in Fig 1B from left to right, application of the scheme involves three major steps: preprocessing of raw images to generate candidates for the structure of interest, selection and calculation features to describe these candidates at both layers of classification, and optimization and training of the two classifiers based on these feature sets.

First, in the preprocessing step, we apply a minimum intensity projection to consolidate dark structures of multi-plane bright-field images into a single image (*MP* in Fig 2B) and use Niblack local thresholding to generate discrete binary particles as potential candidates for the grinder particle (*BW*_0_ in Fig 2B). We employ the Niblack local thresholding procedure in both this and our subsequent cell identification application to robustly segment particles, despite the potential variability in local lighting, texture and background tissue intensity as there would be in different imaging setups (Materials and Exp. Methods). Following initial thresholding, preliminary filtering of the binary particles is then applied to remove segmented regions that are either too small (less than 37.5 μm^2^) or too large (greater than 100 μm^2^) to reduce downstream computation (*BW*_1_ in Fig 2B). The remaining particles are processed through our two-layer classification scheme to detect the presence of the pharyngeal grinder.

Second, in the feature selection step, distinct mathematical descriptors that may help to describe and distinguish the structure of interest are calculated for each layer of classification. In the first layer of classification, intrinsic and computationally inexpensive metrics of the particles are computed and used as features (Fig 2C and S2 Fig) in classification of the grinder shape. These features represent a combination of simple, intuitive geometric features, such as area and perimeter, in addition to higher level measures of the object geometry and invariant moments suitable for shape description and identification [36]. Training and application of a classifier with this feature set eliminates candidates on the basis of intrinsic shape (*BW*_2_ in Fig 2C). However, the resulting false positives in Fig 2D show that the information within these shape metrics is insufficient to distinguish the grinder with high specificity.

To refine the description of the biological structure in the second layer classification, we utilize features that describe the relationship of candidate particles to nearby particles and texture (Fig 2E and S3 Fig). Specifically, we note that the grinder resides inside the terminal bulb of the pharynx, which is characterized by a distinct circular region of muscular tissue (Fig 2A). Based on this observation, we define second layer features based on distributions of particle properties within a circular region around the centroid of the grinder candidate particle (S3 Fig). Noting that the pharyngeal tissue is characterized by textural ranges in the radial direction and relative uniformity in the angular direction, we build features sets describing both the radial and angular distributions the surrounding particles (S3 Fig).

**Fig 3:**
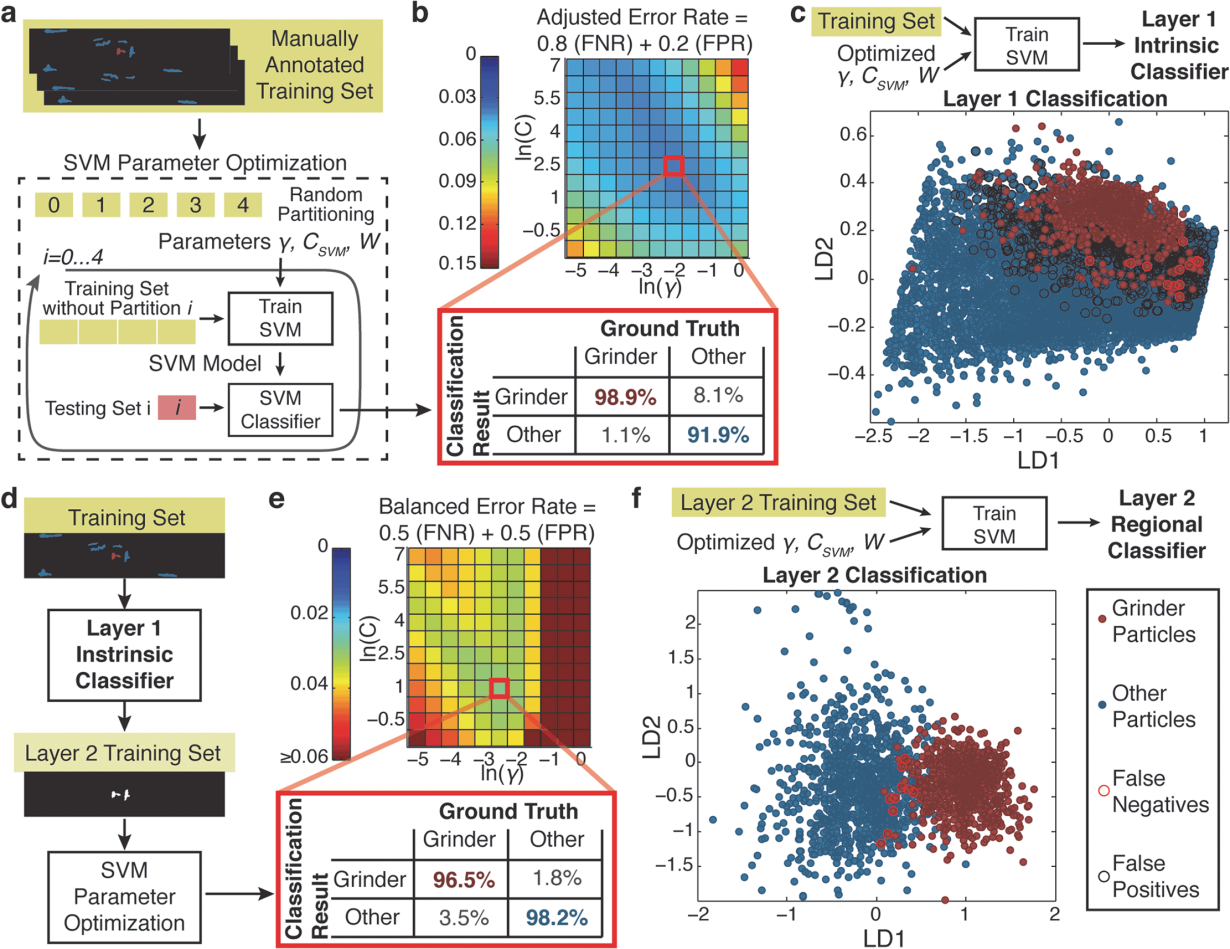
Optimization and training of the two layers of SVM classification for pharyngeal grinder detection. a) To construct the layer 1 classifier with the specified feature set, five-fold cross-validation with a manually annotated training set is first used to optimize SVM model parameters and ensure classification performance. b) Classification performance based on the false positive (FPR) and false negative (FNR) error rates observed in five-fold cross-validation allows selection of an optimal parameter set. c) The full training set and optimized parameters are used to construct the final layer 1 SVM model. Linear projections of the training set features onto two dimensions show that the layer 1 feature set and the optimized SVM model are insufficient for identifying the grinder particle with high specificity. d) The second layer of classification refines the final classification decision and is parameter-optimized using the candidates passed from layer 1 of classification. e) Classification performance based on five-fold cross-validation is used for parameter selection. f) The reduced layer 2 training set and optimized parameters are used to construct the final layer 2 SVM model. Linear projections of layer 2 features for the training set demonstrate the capability of a two layer scheme for the detection of the grinder with both high specificity and sensitivity.

Using the features outlined in Fig 2, each classification step is a mathematical model that is trained to distinguish between structures of interest such as the pharyngeal grinder and irrelevant structures generated represented the textures and boundaries of other tissues in the worm. To allow for supervised training of both the layer 1 and layer 2 classifiers, we annotated a selection of images (n = 1,430) by manually identifying particles that represent the pharyngeal grinder. The classifiers can then be trained to associate properties of the feature sets with the manually specified identity of candidate particles. However, in addition to informative feature selection and the curation of a representative training set, the performance of SVM classification models is subject to several parameters associated with the model itself and its kernel function [34, 48]. Thus, to ensure good performance of the final SVM model, we first optimize model parameters based on five-fold cross-validation on the training set (Fig 3A and 3B, Materials and Methods).

In the parameter selection process, the optimization metric can be designed to reflect the goals of classification in each layer (Fig 3B). In our application, for the first layer of classification, the goal is to eliminate the large majority of background particles while retaining as many grinder particles in the candidate pool as possible for refined classification in the second layer. In other words, we aim to minimize false negatives while tolerating a moderate number of false positives. Therefore, we optimize the SVM parameters via the minimization of an adjusted error rate that penalizes false negatives more than false positives (Fig 3B). We show that with an appropriate parameter selection, the first layer of classification can eliminate over 90% of background particles while retaining almost 99% of the true grinder particles for further analysis downstream (Fig 3B).

To visualize feature and classifier performance, we use Fisher’s linear discriminant analysis to linearly project the 14 layer 1 features of the training set onto two dimensions that show maximum separation between grinder and background particles (Fig 3C). A high degree of overlap between the distributions of the grinder and background particles and high error rates associated with the trained SVM in this visualization suggest that shape-intrinsic features are insufficient to fully describe the grinder structure. Nevertheless, the first layer of classification enriches the true grinder structure candidates in the training set from roughly 6.2% of the original particle set to 40% of the particle set entering into the second layer of classification (Fig 3C). This enriched set of candidate particles is used to optimize and train the second layer of classification in a similar manner (Fig 3D). With appropriate parameter selection, we show that the second layer of classification is capable of identifying the grinder with sensitivity and specificity above 95% (Fig 3E). We train the final layer 2 classifier with the reduced training set and these optimized parameters to yield high classification performance in combination with layer 1 (Fig 3F).

**Fig 4:**
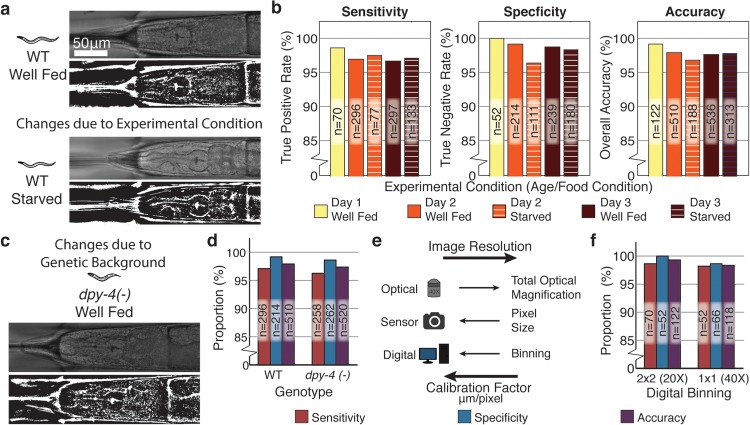
Head versus tail classification using grinder detection is robust to changes in experimental conditions and genetic background. a) Changes in experimental conditions, such as food availability, can alter the bulk morphology and the appearance of worm body in bright field, with potential consequences for classification accuracy. b) Our head versus tail classification scheme maintains sensitivity and specificity at over 95% at different ages and feeding conditions despite these biological changes. c) Genetic changes can also induce changes in bulk morphology and texture of the worm. d) Despite not being represented within the training set, the performance of the classifier is maintained even for mutant worms (*dpy-4 (-)*) with major morphological changes. e) Changes in the optics, camera or acquisition parameters can alter the final resolution of images. f) The inclusion of the calibration metric within feature calculation (S2 Fig and S3 Fig) maintains classifier performance across a two-fold change in image resolution due to alternations in digital binning.

Changes in experimental conditions, the genetic background of the worms under study or changes to the imaging system, can cause significant variation in the features, and thus degrade the classifier performance due to overfitting that fails to take into account experimental variation (Fig 3). To account for this potential variability, we include worms imaged at different ages and food conditions in the training set of images. To validate the utility and efficacy of the resulting classification scheme in a real-life laboratory setting, we analyze its performance on new data sets that were not used in training the classifier. First, in spite of morphological changes due to experimental conditions (Fig 4A), we show the resulting classification scheme operates with consistently high performance in distinguishing the head and the tail of the worm in the new data sets (Fig 4B). Second, while the training set only includes wildtype worms imaged under different conditions, the morphology and texture of the worm is also subject to genetic alteration (Fig 4C). To see whether our classification scheme can accommodate some of this genetic variability, we validate the classification scheme against a mutant strain (*dpy-4(-)*) with large morphological changes in the body of the worm (Fig 4C). Finally, changes in the imaging system can alter the digital resolution of biological structures of interest (Fig 4E). We show that the inclusion of a calibration factor adjusting for the pixel to micron conversion of the imaging system is sufficient for maintain classifier operation across a two-fold change in the resolution of the imaging system (Fig 4F). Thus, this calibrated classification scheme can be easily adapted to systems with different camera pixel formats via the calculation of a new calibration factor.

### Identification of Fluorescently Labeled Cells

An ever-expanding array of fluorescent markers and biosensors [6] has made the identification of specific fluorescent objects and patterns a common biological image processing problem. Although fluorescent staining or tagging techniques can be used to target structures or molecules of interest, they often cannot offer perfect specificity. Furthermore, biological specimens can also include autofluorescent elements that confound the analysis of fluorescent images. Thus, sifting relevant information from fluorescent images can pose non-trivial image processing problems where background fluorescent objects can have similar intensities or spatial locations.

The usage of fluorescent tools in *C*. *elegans* is no exception. Existing toolsets permit fluorescent labeling of different genetic outputs of subsets of cells and tissues. However, fluorescent tags also often label multiple cells, cellular processes or tissue structures that must be distinguished to address specific biological questions. Moreover, *C*. *elegans* exhibits significant gut autofluorescence that varies in intensity and can obscure the identification of fluorescent targets throughout the length of the worm [44]. Here, we demonstrate the use of our framework to address these common challenges in fluorescent image processing, using neuron identification in the worm as a broadly useful example.

**Fig 5:**
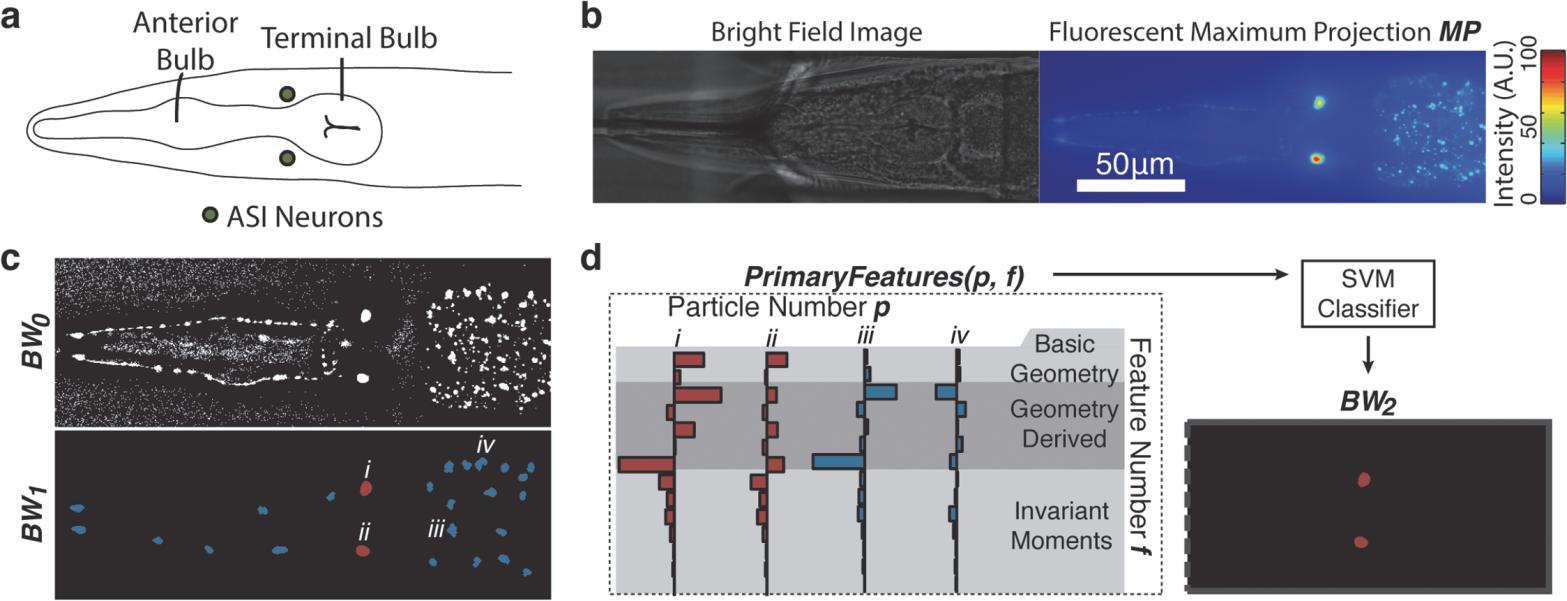
First layer classification for detection of fluorescently labelled neuronal cells demonstrates generalizability of first layer features for particle shape classification. a) Stereotypical positioning of the ASI neuron pair in the head of the worm. Many neuronal cells in the worm are organized as similar pairs near the pharynx. b) Bright field and fluorescent maximum intensity projection showing the appearance and positioning of fluorescently labelled ASI cells in the head of the worm. c) Preprocessing of raw fluorescent images showing binary image after Niblack thresholding (*BW*_0_) and initial filtration of the candidate set by size (*BW*_1_). d) First layer classification of fluorescently labeled neurons shows good generalizability of the first layer feature set developed for pharyngeal grinder detection for classification based on binary particle shape.

We first focus on the identification of the ASI neurons as a stereotypical example of a bilaterally symmetric neuron pair in the worm. Fig 5B shows a corresponding set of bright field and fluorescent images illustrating the positioning of the neuron pair within the head region of the worm. In addition to the cell bodies of interest, the raw fluorescent image also shows cellular processes and autofluorescent granules in the gut of the worm that can confound cell-specific image analysis. Similar to our approach for pharyngeal grinder detection in Fig 2B, we begin building our cell identification toolset via preprocessing of the raw images by maximum intensity projection, Niblack thresholding and preliminary filtering of the resulting candidate particles (Fig 5C, Materials and Exp. Methods). In the selection of features for both layers of classification, we note that the layer 1 feature set we developed for the detection of the pharyngeal grinder can be generally applied to the description of particle shape within other contexts (S2 Fig). Using this feature set, we optimize and train a layer 1 SVM classifier using a manually annotated training set (n = 218) (S4A Fig, Materials and Methods) and show that it is sufficient for identifying cellular regions with relatively high sensitivity and specificity (Fig 5D and S4A Fig).

**Fig 6:**
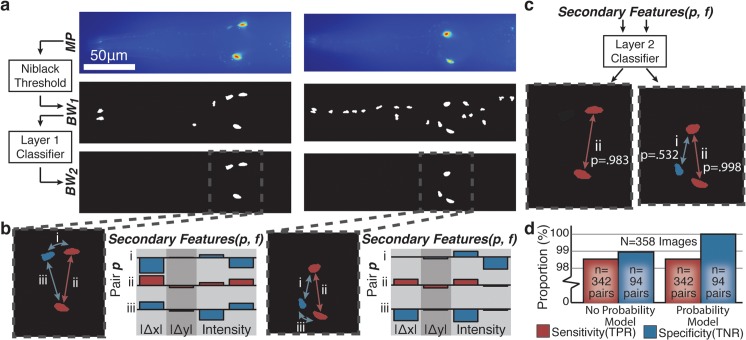
Second layer classification for neuron pair detection. a) The first layer of classification is insufficient for rejection of all background particles. b) The reduced candidate set from the first layer of classification is used to form candidate cell pairs with feature sets describing their relative positioning and intensities. c) Although classification based on these features is sufficient for accurate cell pair detection in the majority of cases (left), multiple potential cell pairs are sometimes classified within the same image (right). d) Incorporating probability estimates (shown in panel c) into the SVM model and selecting the most likely cell pair eliminates these false positives and increases the specificity of the classifier.

While the first layer of classification is effective at eliminating the large majority of background particles, variable background intensity within the tissues surrounding the neurons can generate confounding binary particles that pass layer 1 classification (Fig 6A). To make a final identification of a true cell pair, we apply a second layer of classification based on the relational properties of potential pairs of particles that pass layer 1 classification (Fig 6B and S5 Fig). To construct our layer 2 classifier, we optimize and train an SVM model based on these pairwise relational features (S4B Fig). We note that while the relational features we utilize are computationally simple, embedding relational features on the second layer of classification dramatically reduces the size of the paired candidate set. For example, for detection of cell pairs amongst n particles, there are $\left(\begin{array}{c} n\\ 2 \end{array}\right)=\frac{n!}{2\left(n-2\right)!}$ possible candidate pairs that require feature calculation.

Validating the resulting cell pair classifier against new test images, we find robust single cell-pair detection in the majority of cases (Fig 6C, left). However, in a minority of cases, multiple candidate pairs are identified as potential neuron pairs in each image (Fig 6C, right). This is a common scenario as many promoters used in transgene markers are not necessarily specific to a single class of cells. In this case, the probability estimates from the SVM classifier [37, 49] along with the selection of the most likely candidate in images with multiple positive classification results is used to eliminate these false positives. This boosts the specificity of the classifier without compromising the high sensitivity (Fig 6D). This additional step incorporates the real-world constraint that, at most, one cell pair exists in each valid image and resolves any conflicts that may arise in direct classification.

**Fig 7:**
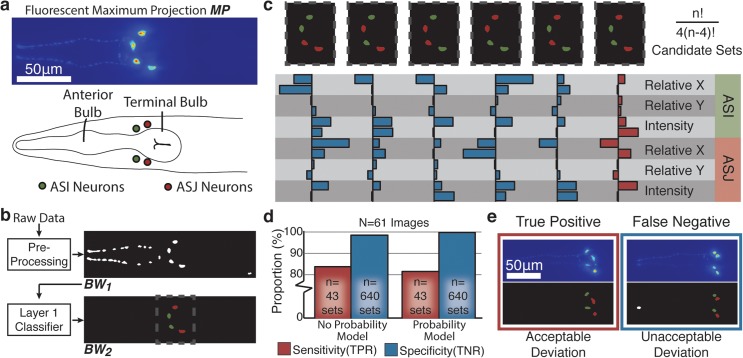
Second layer classifier for cell pattern recognition and identification. a) Representative maximum intensity projection and schematic representation of the two neuron pairs in which an insulin-like peptide is expressed. b) The modularity of our scheme permits the preprocessing and layer 1 classification components from neuron pair detection to be re-used for the recognition and identification of these neuron pairs. c) To identify the pattern with the appropriate cell identifications, properties for all possible combinations and arrangements of the layer 1 candidates are calculated. Here, all six such candidate sets for 4 candidate particles are shown. d) Validation of the SVM classifier trained with these features shows high specificity but only moderate sensitivity. e) The lower sensitivity observed for this classification scheme is mainly due to the limit ability to accommodate biological deviations from the stereotypical arrangement of the neurons while still maintaining high specificity.

To demonstrate the ability of our framework to detect more complex cellular arrangements, we use the expression pattern of a worm insulin-like peptide gene (*ins-6*) in two bilaterally symmetric neuron pairs (Fig 7A) [50]. In this case, the specificity offered by the *ins-6* promoter is insufficient to offer full cell specificity, requiring the identification of different cells from the raw fluorescent image. Taking advantage of our modular two-layer architecture, we reuse the preprocessing and first layer classification tools that we have already constructed to identify a small number of cell-shaped objects shown in Fig 7B. To detect the tetrad of cells with specificity for the ASI and ASJ neurons, we construct a relational feature set based on combinations of neuron pairs (S6 Fig). As shown in Fig 7C, accounting for both correct cell pair identification and non-repetition of individual cells within the tetrad set, there are $\left(\begin{array}{c} n\\ 2 \end{array}\right)\left(\begin{array}{c} n-2\\ 2 \end{array}\right)=\frac{n!}{4\left(n-4\right)!}$ tetrad sets that require feature calculation. Our two-layer architecture is therefore essential for the construction of such relational feature sets with larger numbers of targets. Without layer 1 classification, description of such complex sets quickly becomes intractable: even 10 candidate particles generates 1,260 different possible tetrad sets for feature calculation.

To construct a new problem-specific layer 2 classifier based on relationships within these tetrad candidates, we optimize and train a SVM model based on a manually annotated training set (n = 324) (S4C Fig). Subsequent validation of our two-layer classifier against new test images shows that the two-layer classification scheme operates with higher specificity but lower sensitivity in comparison to our single cell-pair classification problem (Fig 7D). Further analysis of the classifier performance within the test set of images shows that this lower sensitivity is mainly due to more degrees of freedom for variability associated with this particular image processing problem. As shown in Fig 7E, while the second-layer classifier accommodates some deviation from the stereotypical arrangement of the neurons shown in Fig 7A (positive identification on the left), there is a trade-off between maintaining specificity and sensitivity (rejecting larger deviations as illustrated by the negative identification on the right). If the stringency is important, i.e. maintaining specificity and reducing misidentification rate, the users would have to tolerate a small amounts of false negatives. Users would need to determine a comfortable level of rejection rate for each specific problem to tune the classifier.

## Discussion

We have demonstrated the flexibility and computational benefits of our two-layer architecture in handling two disparate image recognition problems. Using our pipeline, we have developed two specific solutions addressing common image processing problems for the *C*. *elegans* community. Our contribution of a ready-to-use head-versus-tail classification scheme under bright-field imaging enables automated high-resolution imaging and stimulus application in a large range of biological experiments in the worm. Our neuronal cell pair identification application forms the basis for approaching the general problem of cell-specific information extraction within a multicellular context such as the worm. Together, these specific tools permit automated visual dissection of the multicellular worm at different resolutions that range from the targeting of rough anatomical regions to cell-specificity.

In addition to the immediate utility of the two examples provided in this work, they are also representative of two classes of problems that are commonly found in biological image processing. The detection of the pharyngeal grinder demonstrates a general class of problems where discrete structures are distinguished by both their intrinsic shape and the characteristics of their local environment. The entire framework, including the feature sets, developed and documented for this problem can be applied to the recognition of other discrete structures including subcellular organelles such as nuclei, specific cell types and tissue structures. The detection of single and multiple cell pairs extends the analysis to stereotypical formations of objects. The feature sets documented here for analyzing paired objects is directly applicable to the analysis of many symmetrical structures that arise in biology, such as in the nervous system. However, with some modification, similar features can be applied to the analysis of different patterns that may arise in specific biological processes such as development. Finally, the preprocessing modules developed for these two applications demonstrate the ability to segment out objects of different intensities from both bright-field and fluorescent imaging and are applicable to many other problem sets.

Our two specific applications also highlight the effectiveness of our algorithm in segregating complex image recognition problems in both a computationally effective and conceptually convenient manner. In the detection of the pharyngeal grinder, two-layer construction eliminated the need to compute a large set of regional descriptors by associating them with the second layer of classification and therefore a smaller candidate set. In comparison with direct calculation of all features in a single layer of classification, the two-layer architecture employed in this work reduced average total computational time by a factor of two (S7 Fig). In cell identification, reserving relational properties for the second-layer of classifications dramatically reduced the number of pairs or sets for which relationships must be described. In this case, the computational time savings associated with the two-layer architecture increases with the complexity of the second-layer relationships and can result in large, roughly six-fold speed improvements in the case of two cell pair identification (S8 Fig). In addition to these computational benefits, our two applications also demonstrate that the segregation of intrinsic and secondary or extrinsic properties of a structure onto two layers of classification reserves many problem-specific features for the second layer and renders the first layer feature set generalizable. In addition, we have demonstrated that by incorporating a calibration factor to normalize feature calculation, these classifiers can be adapted to different optical systems and sensor configurations with only the modification of the calibration factor itself (Fig 4E and 4F and S1 Table).

In both layers of classification, we adopt a supervised learning approach that depends upon human annotation of training sets of data. This approach imposes user-defined structure onto the data-extraction problem and promotes familiarization with the condition and fundamental limitations on the information content of imaging datasets [29, 51]. Moreover, having a small set of manually annotated images allows for the assessment of the reliability of the final analysis [29]. Thus, the user exercises control over higher level problem structure including the formulation of the overall classification question, the choice of the type of candidates and the features used. However, to constrain the construction of the solution, we present a specific workflow with integrated computational techniques that bypass much of the manual guesswork. Annotation and calculation of quantitative descriptors about particle or pixel candidates captures multivariate information about different structures. The use of this multivariate information with a classification model such as SVM obviates the need for manually assessing rectilinear thresholds for classification. Moreover, the performance of our classifiers demonstrate that the potentially nonlinear, multi-dimensional classification provided by SVM prove more powerful than rectilinear thresholding of individual features or dimensionality reduction techniques (Fig 3C and 3F). Overall, our proposed methodology provides a pipeline that streamlines and formalizes the image processing steps after the annotation of a training set.

Finally, while utility of our framework will require feature selection and training for each particular application, the modularity and architecture of our framework permits aspects of the specific tools we have developed here to be reused. In general, the construction of our classification scheme affords layer 1 classifiers more general applicability. For example, we have demonstrated the generalizability of our layer 1 feature set for binary particle classification with re-training for the identification of different shapes. The layer 1 classifier constructed in our cell identification scheme can also be reused for the classification of different downstream cellular arrangements. Even for the second layer of classification, where feature sets are problem-specific, we have provided examples of both regional and relational feature set constructions that can form the basis of feature sets for other problems.

### Conclusion

Beyond the specific applications we discuss here, we envision that our methodology can be a powerful way to tackle a broad range of biological image processing problems. For instance, we consider our scheme to be a generalization of the previously reported application of SVMs towards the understanding of synaptic morphology in *C*. *elegans* [24]. In this application, individual pixels within the image form the pool of candidates for potential synaptic pixels in the first layer classification. The second layer of classification then refines this decision on the basis of relational characteristics between candidates. Here, we formalize this classification approach and demonstrate that it can be adapted towards detection of disparate structures imaged under different imaging modalities. The imaging processing approach we present here has inherent structural advantages in terms of conceptual division, modularization and computational efficiency and demonstrates the application of a powerful supervised learning model to streamline biological image processing. We thus envision that our methodology can form the basis for detection algorithms for structures ranging from the molecular to the tissue or organismal level under different experimental methodologies.

## Materials and Methods

### Worm Maintenance and Culture

*C*. *elegans* worms used in this study were maintained and cultured according to standard techniques [52]. Briefly, populations of worms were allowed to reach reproductive maturity and lay eggs on NGM agar media overnight. Age-synchronized worms were then obtained by washing free-moving worms off of the agar plate, allowing the remaining eggs to hatch for one hour and then washing the resulting L1 stage larvae off of the plate. Age-synchronized L1 worms were then transferred onto new NGM plates seeded with OP50 *E*. *coli* bacteria as a standard food source and grown until the desired age for imaging. To avoid over-crowding and food depletion, adult worms were transferred onto new plates daily. For starvation experiments, worms were transferred onto fresh NGM plates lacking a bacterial food source the day before imaging.

*C*. *elegans* strains used in these studies were wild-type N2 worms, QH3833 *dpy-4*(*e1166*), QL296 *drcSi89[pdaf-7*::*GFP; unc-119(+)]* and QL617 *drcSi68[unc-119(+); Pins-6*::*mCherry]II; gjIs140[dpy-20(+); gpa-4*::*GFP]*.

### Microfluidics and Image Acquisition

We use standard soft lithographic techniques to produce polydimethylsiloxane (PDMS) imaging devices similar to those previously described [24, 38]. For automated imaging, worms are washed off of NGM plates using S Basal buffer and introduced via pressure injection into the microfluidic device. Sequential activation of pressure sources driving liquid delivery and on-chip pneumatic valves is then used to drive individual worms within the device for imaging.

Images were collected either on a Leica DMI 6000B microscope with a Hamamatsu Orca D2 camera and a 40X oil objective or on an Olympus IX-73 microscope with a Hamamatsu Flash 4.0 camera and a 40X oil objective. Relevant specifications and calibration metrics for these set-ups can be found in S1 Table. Although not strictly necessary, for generalizability in cases where the center of focus is adjusted to specific fluorescent targets and do not capture the pharynx well, a sparse three plane z-stack with a 15μm step size is used for bright field image acquisition. To fully capture neuronal cells, a dense z-stack was collected through the body of the worm. For fluorescence imaging of the single neuron pair in QL296, a 0.4μm step size was used over a 60μm thick volume. For fluorescence imaging of multiple neurons pairs in QL617, a 1μm step size was used over a 100μm thick volume.

### Image Analysis and Computational Tools

We use custom MATLAB code to perform all image preprocessing and feature extraction steps and enable the construction and testing of our classification schemes. In preprocessing, the three dimensional information in the acquired z-stacks were either maximum or minimum projected onto a single two-dimensional image for further processing. For bright-field images, a minimum projection with respect to z was utilized to accentuate the appearance of dark objects throughout the stack. Conversely, for fluorescence images, a maximum projection was utilized to accentuate the appearance of bright objects throughout the stack:

$$MP_{BF}\left(x_{i},y_{i}\right)=\underset{z_{i}}{\min}\left(I\left(x_{i},y_{i},z_{i}\right)\right),MP_{FLUO}\left(x_{i},y_{i}\right)=\underset{z_{i}}{\max}\left(I\left(x_{i},y_{i},z_{i}\right)\right)$$

In order to generate binary particles for classification, we use a local thresholding algorithm that uses information about the mean and variability of pixel intensities within a local region around a pixel:

$$\begin{array}{c} BW_{BF}\left(x_{i},y_{i}\right)=MP_{BF}\left(x_{i},y_{i}\right)\leq \mu_{local}-k\sigma_{local},\\ BW_{FLUO}\left(x_{i},y_{i}\right)=MP_{FLUO}\left(x_{i},y_{i}\right)\geq \mu_{local}+k\sigma_{local} \end{array}$$

*μ*_*local*_ and *σ*_*local*_ are the means and standard deviations of all pixel values that fall within a square region of width 2*R* + 1 centered around the pixel of interest *x*_*i*_, *y*_*i*_ and *k* is a parameter specifying the stringency of the threshold. *μ*_*local*_ and *σ*_*local*_ can be derived using standard image filtering with a binary square filter *h*(*x*_*i*_, *y*_*j*_) of width 2*R* + 1:

$$\begin{array}{c} \mu_{local}=\frac{1}{{\left(2R+1\right)}^{2}}filter\left(MP\left[i,j\right],h\left[i,j\right]\right)=\frac{1}{{\left(2R+1\right)}^{2}}\sum_{m=0}^{M}\sum_{n=0}^{N}MP\left[i,j\right]h\left(i-m,j-n\right)\\ \sigma_{local}=\sqrt{\frac{1}{{\left(2R+1\right)}^{2}}filter\left({\left(MP\left[i,j\right]-\mu_{local}\left[i,j\right]\right)}^{2},h\left[i,j\right]\right)} \end{array}$$

Using local mean and standard deviation information in the binary decision affords robustness against local background intensity and texture changes.

The width of the local region, *R*, can be roughly selected on the basis of the size scale of the structure of interest. In accordance with the size scales of the pharyngeal structure and individual neurons, we use *R* = 15*μm* for detection of the pharyngeal grinder and *R* = 5*μm* for fluorescent cell segmentation.

The parameter *k* can be roughly selected by visual inspection of segmentation results. We use *k* = 0.75 for our bright field application and *k* = 0.85 for our fluorescence application. Individual candidate particles in the resulting binary image are defined as groups of nonzero pixels that are connected to each other via any adjacent of diagonal pixel (8-connected). We note that changes in *k* can alter the size of segmented particles and the connectivity of segmented particles. Particularly in bright field, where the contrast mechanism lacks specificity, decreases in *k* can cause particles to merge via small bridges of dark texture. In order to build in some robustness against changes in *k* and background texture in these scenarios, we perform a form of a morphological opening operation after thresholding to remove small bridges that may arise between otherwise distinct particles. To do this, we perform a morphological erosion with a small circular structuring element followed by a morphological dilation with a smaller structuring element [53].

In order to fully capture both intrinsic and secondary characteristics of biological structures, we calculate distinct sets of features for two layers of classification. The first layer, which delineates structures of interest from other structures on the basis on its intrinsic geometric properties, is generally applicable to particle classification problems and is used for both the bright field and fluorescent structure detection outlined here. Details and equations for the calculation of the 14 features for layer 1 classification can be found in S2 Fig.

Secondary characteristics of biological structures describe the context in which structures exist and their relationship to other structures. Due to the large variability in the secondary characteristics of biological structures, a generic set of features is not necessarily attainable or desirable due to concerns for computational efficiency. Rather, secondary features can be derived via a mathematical description of empirical observations of important structural properties. In the case of pharyngeal grinder detection, the secondary features are regional, forming a description of the image context in which the grinder structure resides. The form of the features is based on an empirical understanding of this structural context and full details and equations for the calculation of the 34 features in layer 2 of the bright field classifier can be found in S3 Fig. In the case of cell pair detection, the secondary features are mostly relational, describing how particles from layer 1 of classification may or may not exist as pairs on the basis of both positioning and intensity. Second layer features for cell pair detection can be found in S5 Fig.

We do briefly note that we scale all calculated features using a calibration factor, *C*, derived from specifications of both the optics and sensors that form the imaging system:

$$C=\frac{\left(Sensor\;Pixel\;Size\right)\left(Binning\right)}{\left(Optical\;Magnification\right)}$$

The use of this calibration system renders the trained classifier relatively invariable to small changes in the imaging set-up via conversion of all features into real units. Calibration factors for all imaging systems and configurations used here can be found in S1 Table.

To implement discrete classification steps using support vector machines, we use the LIBSVM library, which is freely available for multiple platforms including MATLAB [37]. For general performance, we train use a Gaussian radial basis function kernel for all of our trained classifiers [48]. To ensure performance of the SVM model for our datasets, we optimize the penalty or margin parameter, *C*_*SVM*_, and the kernel parameter, *γ*, for each training set using the five-fold cross-validation performance of the classifier as the output metric. For efficient parameter optimization, we start with a rough exponential grid search (Fig 3B and 3D and S4 Fig) and refine parameter selection with a finer grid search based on these results. To adjust for the relative proportions of positive and negative candidates in unbalanced training sets (Fig 3C), we also adjust the relative weight, *W*, of the classes according to their representation in the training set while training [37]. Additionally, we perform a small grid search for the optimal weighting factor to fully optimize the following performance metric. Probability estimates for single and multiple neuron pair identification are derived according to the native LIBSVM algorithm [37].

For visualization of the high dimensionality feature sets (Fig 3C and 3F), we apply Fisher’s linear discriminant analysis [54]. The two projection directions are chosen to be the first two eigen vectors of:

$$\begin{array}{c} S_{w}^{-1}S_{B}\\ S_{B}=\left(\mu_{1}-\mu_{2}\right){\left(\mu_{1}-\mu_{2}\right)}^{T}\\ S_{w}=S_{1}+S_{2},\;S_{i}=\sum_{x\in class\;i}\left(x-\mu_{i}\right){\left(x-\mu_{i}\right)}^{T} \end{array}$$

*S*_*B*_ is a measure of inter-class separation and *S*_*W*_ is a measure of intra-class scatter.

## Supporting Information

S1 Fig Images Collected with Standard Agar Pad Techniques Can Also Be Subjected to the same Analysis for Identification of the Grinder. a, b and c show three representative images of day 2, well-fed adult worms acquired using standard agar pad imaging techniques. The intermediate outputs of grinder detection (*MP*, *BW*_0_, *BW*_1_, *BW*_2_, *BW*_3_) show the minimally projected image, the binary image after thresholding, the initial particle candidate set, the candidate set after the first layer of classification and the final particle set after the second layer of classification, respectively. The same process developed for head versus tail analysis on microfluidic chip robustly identifies the grinder structure in these conventionally acquired images.
(TIF)

S2 Fig Robust Descriptors for Binary Particle Shape for Layer 1 of Classification Scheme. a) Table of 14 features for binary shape description including low-level geometric descriptors, more complex derived measures of geometry and invariant moments. b) Diagram of binary particle indicating variables used for feature definition. c) Illustration and example of defining and calculating the perimeter of an irregular particle based on pixel connectivity. d) Illustration and example of the convex hull of a binary particle.
(TIF)

S3 Fig Regional Descriptors for Structural Detection of the Pharyngeal Grinder. a) Diagram of the region of interest around a grinder particle showing changes in texture and particle density along radial partitions. b) Diagram of the region of interest around a grinder particle distinguishing individual particles using different colors and showing particle distributions along angular partitions. c) Table of 34 features used to describe regional characteristics of the grinder particle for the second layer of classification.
(TIF)

S4 Fig Parameter selection for the first and second layer classifiers in neuron pair identification. Optimized parameters for the first layer classifier (a), the second layer single pair classifier (b) and the second layer two pair classifier (c) show considerable variability, reinforcing the need for case-specific parameter optimization.
(TIF)

S5 Fig Relational features for pairs of neurons. a) Maximum intensity projection (*MP*) and binary image (*BW*_2_) showing candidate particles after the first layer of classification with relevant axes and regions labeled. b) Identification of possible pairs for feature calculation and schematic of an example feature set for one pair. c) Table of the four relational features used to describe cell pair patterns.
(TIF)

S6 Fig Relational features for multiple cell pair detection and identification. a) Maximum intensity projection (*MP*) and binary image showing candidate particles after layer 1 classification (*BW*_2_) with relevant axes and regions labeled. b) Enumeration of the possible neuron pairs and the possible sets of neuron pairs with correct distinction between the ASI and ASJ pairs. c) Schematic showing the frame of reference (*X*_*C*_, *Y*_*C*_) for the calculation of the relative location of each neuron and the intensities of the neurons within two particular sets. d) Table showing that 6 properties are calculated for each neuron pair, resulting in a total of 12 relational features to identify the tetrad of neurons.
(TIF)

S7 Fig Computational time savings associated with two-layer classification architecture for head versus tail detection. a) Schematic comparisons of the two-layer, serial classification architecture employed in this work and an equivalent single-layer, parallel classification architecture used for time comparisons. b) Comparison of process-specific and total time requirements for the two-layer and equivalent one-layer architectures. Reducing second-layer feature calculations using the two-layer scheme results in over a two-fold reduction in total classification time. All times are based on performance on MATLAB 2013b running on a quad core processor at 3.50 GHz.
(TIF)

S8 Fig Computational time savings associated with two-layer classification architecture for cell identification. a) Comparison of process-specific and total time requirements for the two-layer and equivalent one-layer architectures when applied to single neuron pair detection. b) Comparison of process-specific and total time requirements for the two-layer and equivalent one-layer architectures when applied to the identification of two distinct neuron pairs. All times are based on performance on MATLAB 2013b running on a quad core processor at 3.50 GHz.
(TIF)

S1 Table Calculation of the calibration metric for common changes in the imaging system and acquisition parameters. Setups used for this study are highlighted.
(TIF)
