# Supplementary figures and images for: Automated Processing of Imaging Data through Multi-tiered Classification of Biological Structures Illustrated Using Caenorhabditis elegans
Source: PLoS Comput Biol. 2015 Apr 24;11(4):e1004194. doi: 10.1371/journal.pcbi.1004194 (PMC4409145; doi:10.1371/journal.pcbi.1004194)

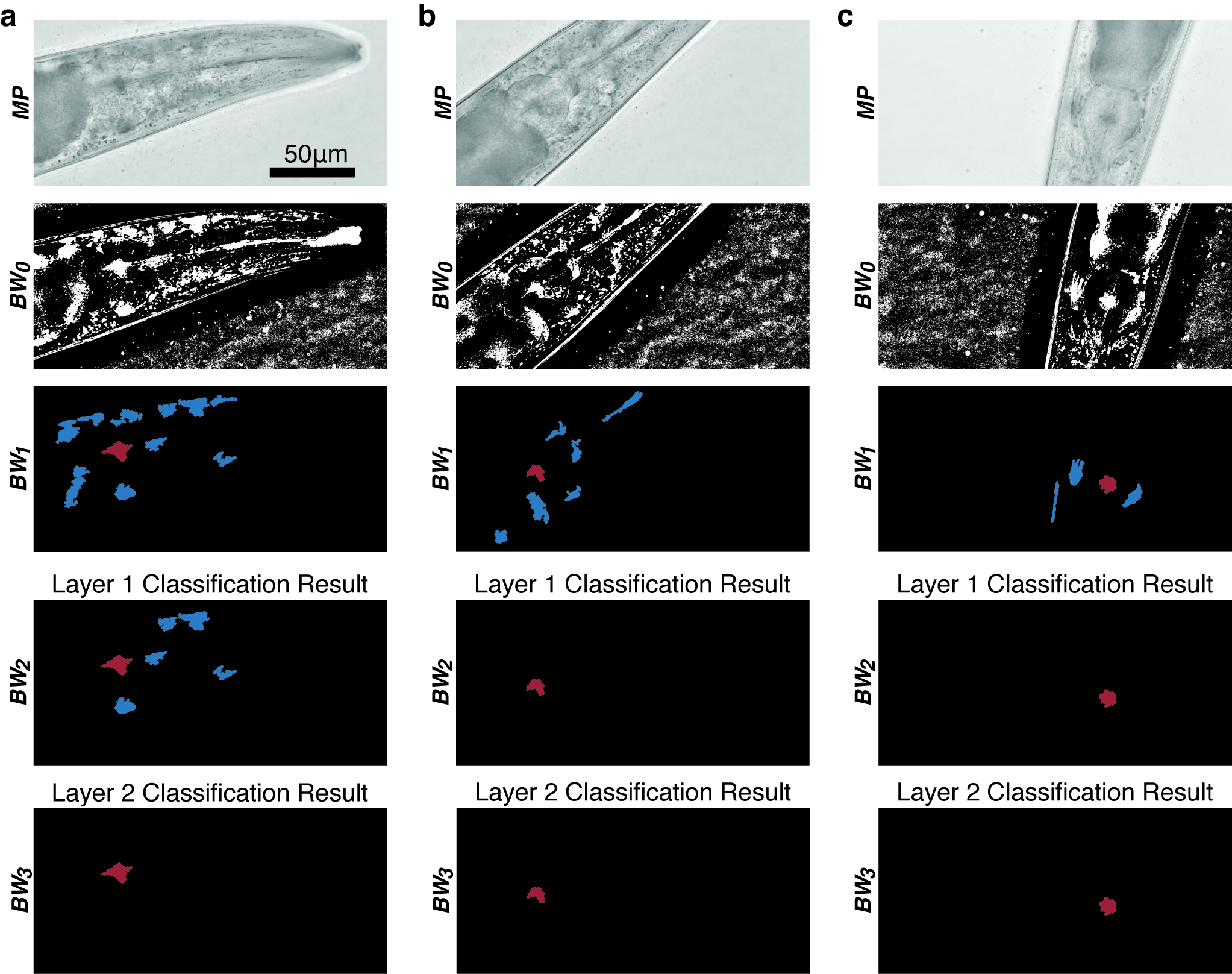

Supplement: S1 Fig — a, b and c show three representative images of day 2, well-fed adult worms acquired using standard agar pad imaging techniques. The intermediate outputs of grinder detection (MP, BW 0, BW 1, BW 2, BW 3) show the minimally projected image, the binary image after thresholding, the initial particle candidate set, the candidate set after the first layer of classification and the final particle set after the second layer of classification, respectively. The same process developed for head versus tail analysis on microfluidic chip robustly identifies the grinder structure in these conventionally acquired images. (TIF) [file pcbi.1004194.s001.tif]

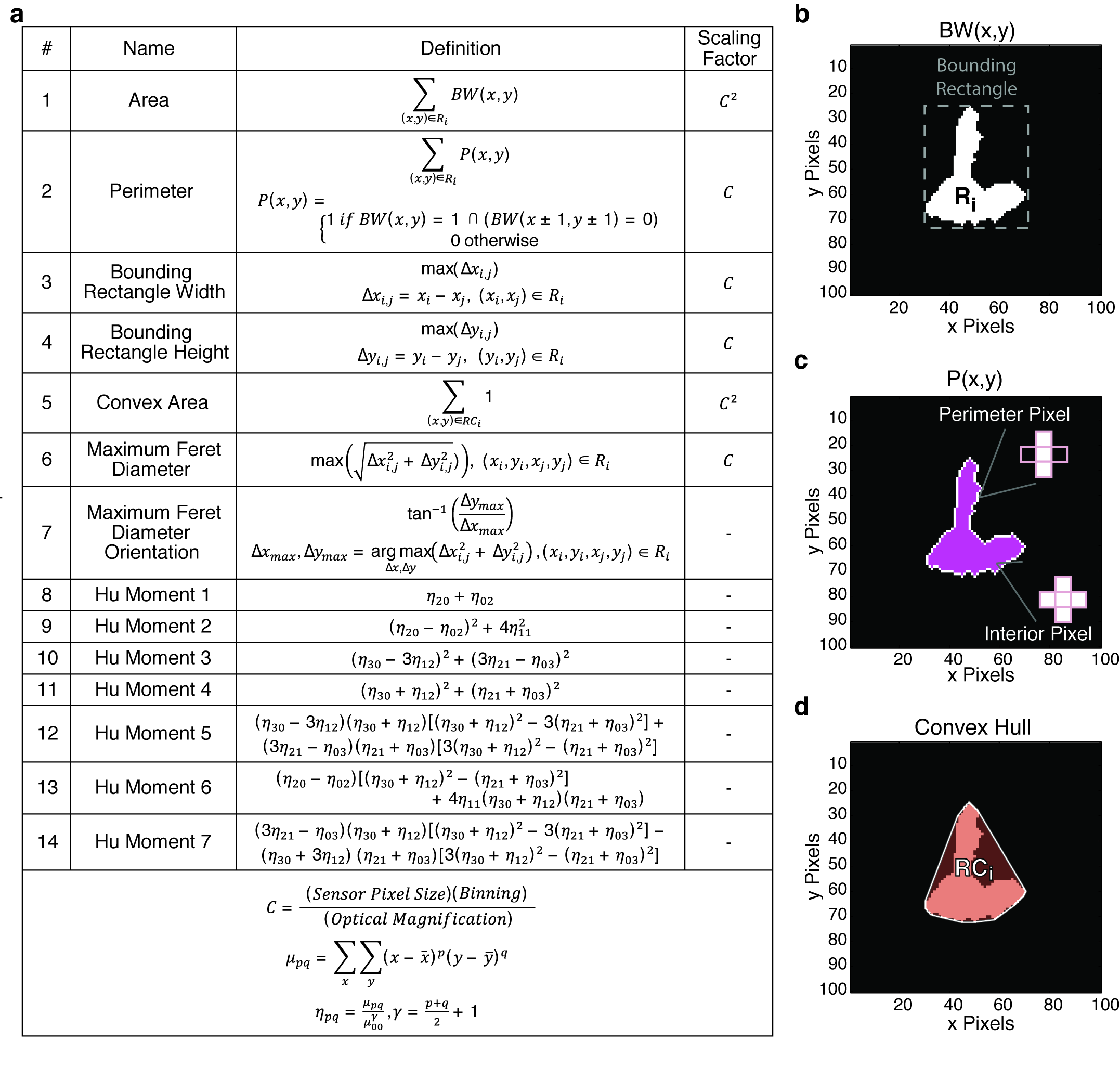

Supplement: S2 Fig — a) Table of 14 features for binary shape description including low-level geometric descriptors, more complex derived measures of geometry and invariant moments. b) Diagram of binary particle indicating variables used for feature definition. c) Illustration and example of defining and calculating the perimeter of an irregular particle based on pixel connectivity. d) Illustration and example of the convex hull of a binary particle. (TIF) [file pcbi.1004194.s002.tif]

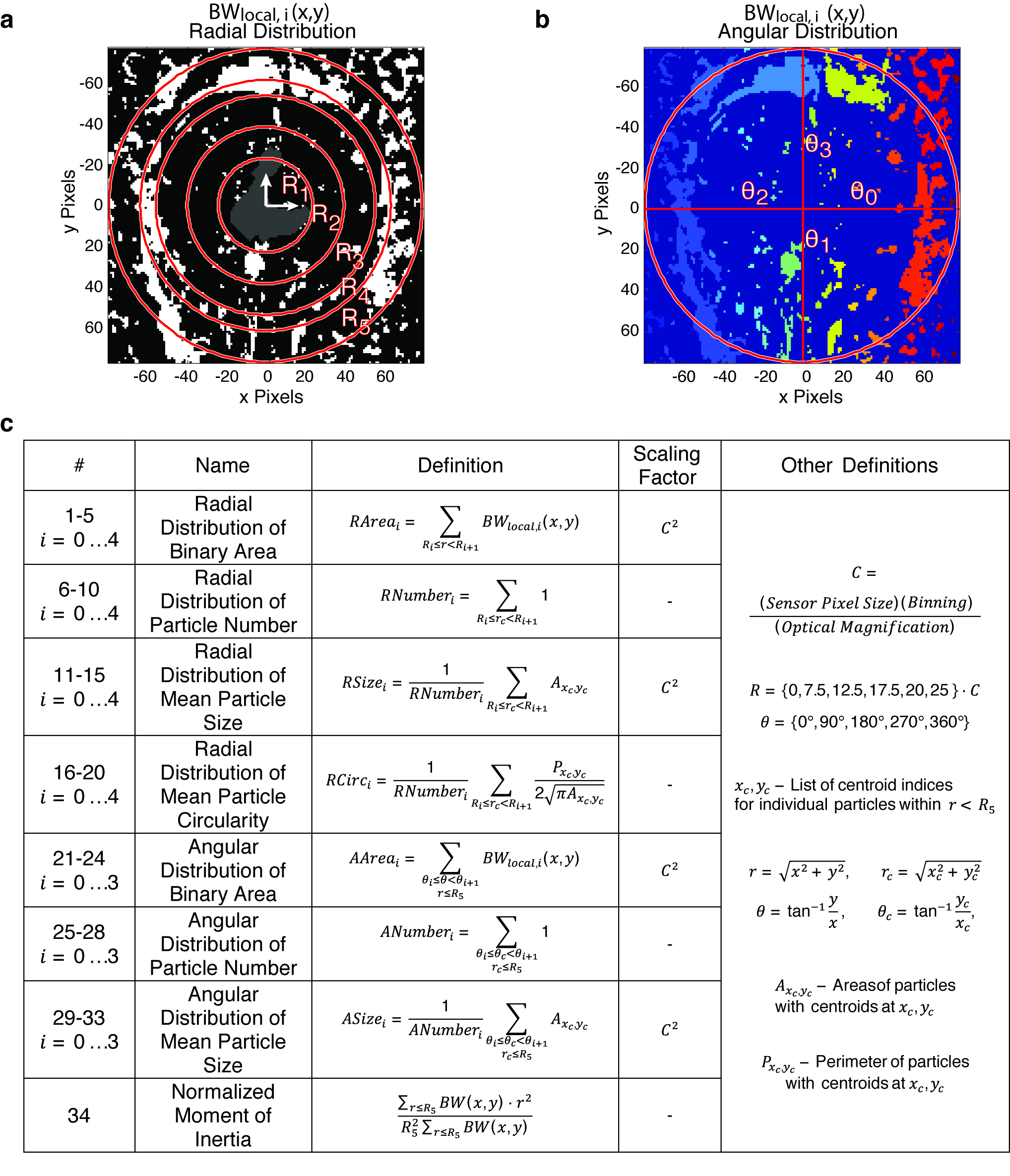

Supplement: S3 Fig — a) Diagram of the region of interest around a grinder particle showing changes in texture and particle density along radial partitions. b) Diagram of the region of interest around a grinder particle distinguishing individual particles using different colors and showing particle distributions along angular partitions. c) Table of 34 features used to describe regional characteristics of the grinder particle for the second layer of classification. (TIF) [file pcbi.1004194.s003.tif]

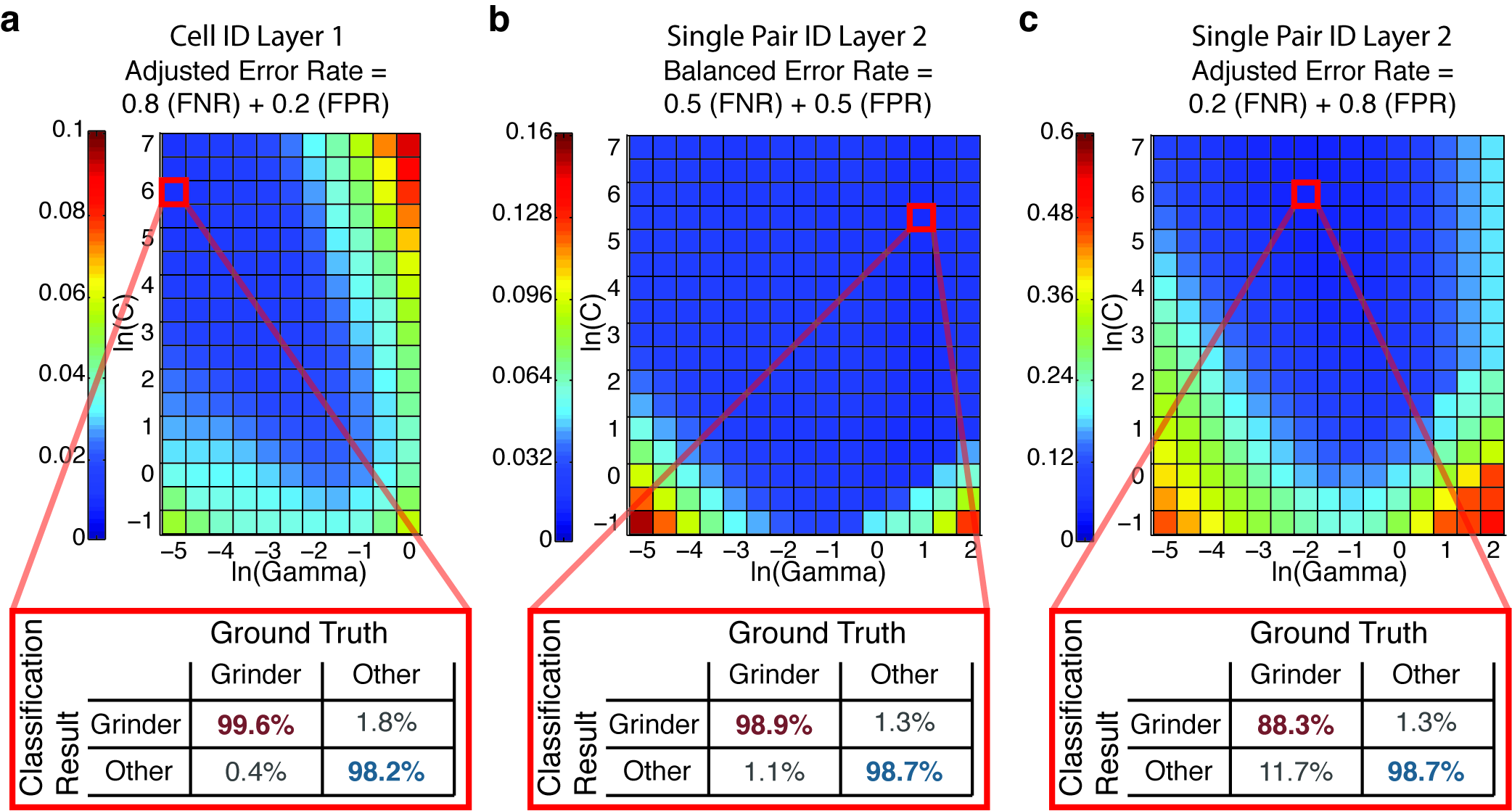

Supplement: S4 Fig — Optimized parameters for the first layer classifier (a), the second layer single pair classifier (b) and the second layer two pair classifier (c) show considerable variability, reinforcing the need for case-specific parameter optimization. (TIF) [file pcbi.1004194.s004.tif]

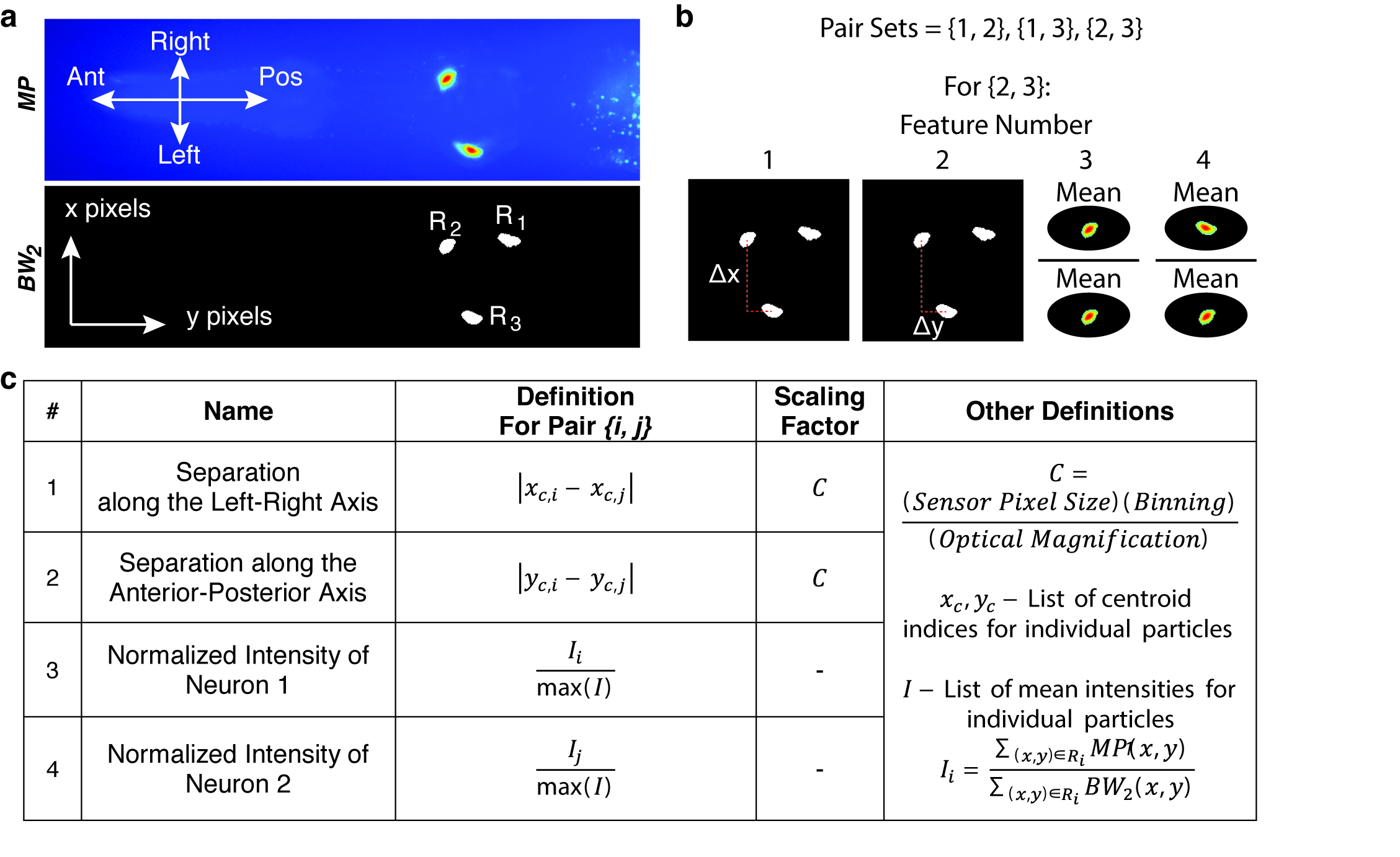

Supplement: S5 Fig — a) Maximum intensity projection (MP) and binary image (BW 2) showing candidate particles after the first layer of classification with relevant axes and regions labeled. b) Identification of possible pairs for feature calculation and schematic of an example feature set for one pair. c) Table of the four relational features used to describe cell pair patterns. (TIF) [file pcbi.1004194.s005.tif]

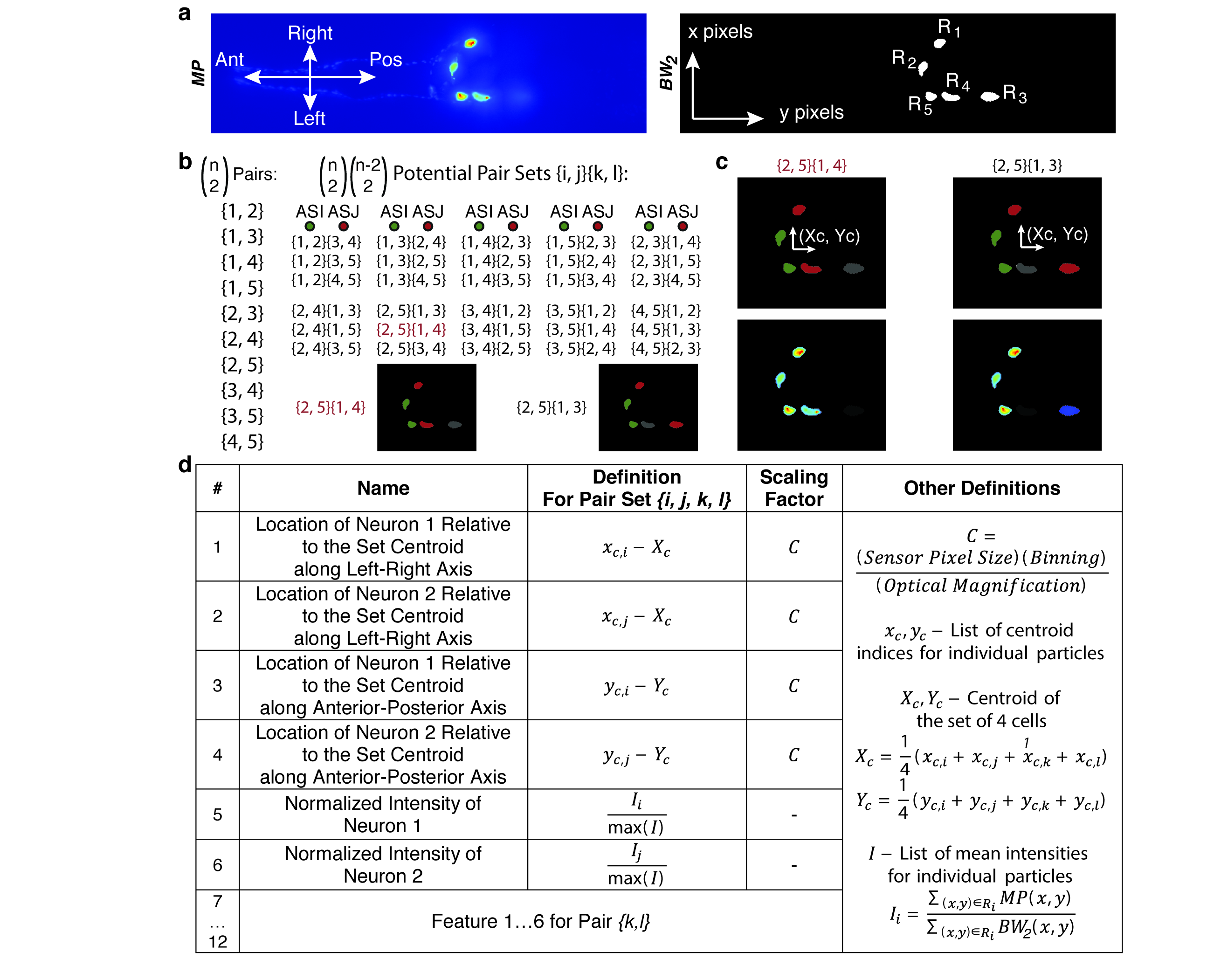

Supplement: S6 Fig — a) Maximum intensity projection (MP) and binary image showing candidate particles after layer 1 classification (BW 2) with relevant axes and regions labeled. b) Enumeration of the possible neuron pairs and the possible sets of neuron pairs with correct distinction between the ASI and ASJ pairs. c) Schematic showing the frame of reference (X C, Y C) for the calculation of the relative location of each neuron and the intensities of the neurons within two particular sets. d) Table showing that 6 properties are calculated for each neuron pair, resulting in a total of 12 relational features to identify the tetrad of neurons. (TIF) [file pcbi.1004194.s006.tif]

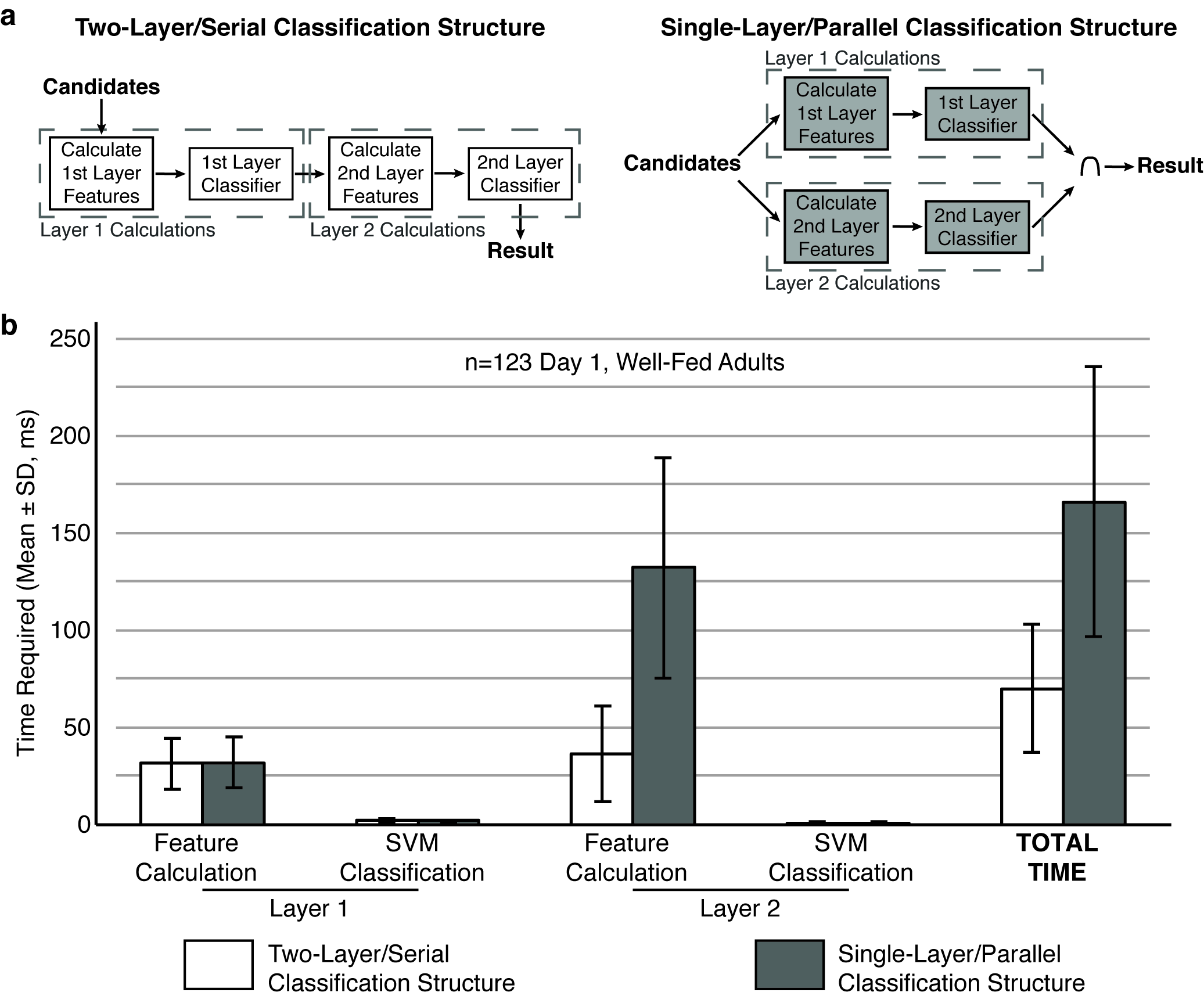

Supplement: S7 Fig — a) Schematic comparisons of the two-layer, serial classification architecture employed in this work and an equivalent single-layer, parallel classification architecture used for time comparisons. b) Comparison of process-specific and total time requirements for the two-layer and equivalent one-layer architectures. Reducing second-layer feature calculations using the two-layer scheme results in over a two-fold reduction in total classification time. All times are based on performance on MATLAB 2013b running on a quad core processor at 3.50 GHz. (TIF) [file pcbi.1004194.s007.tif]

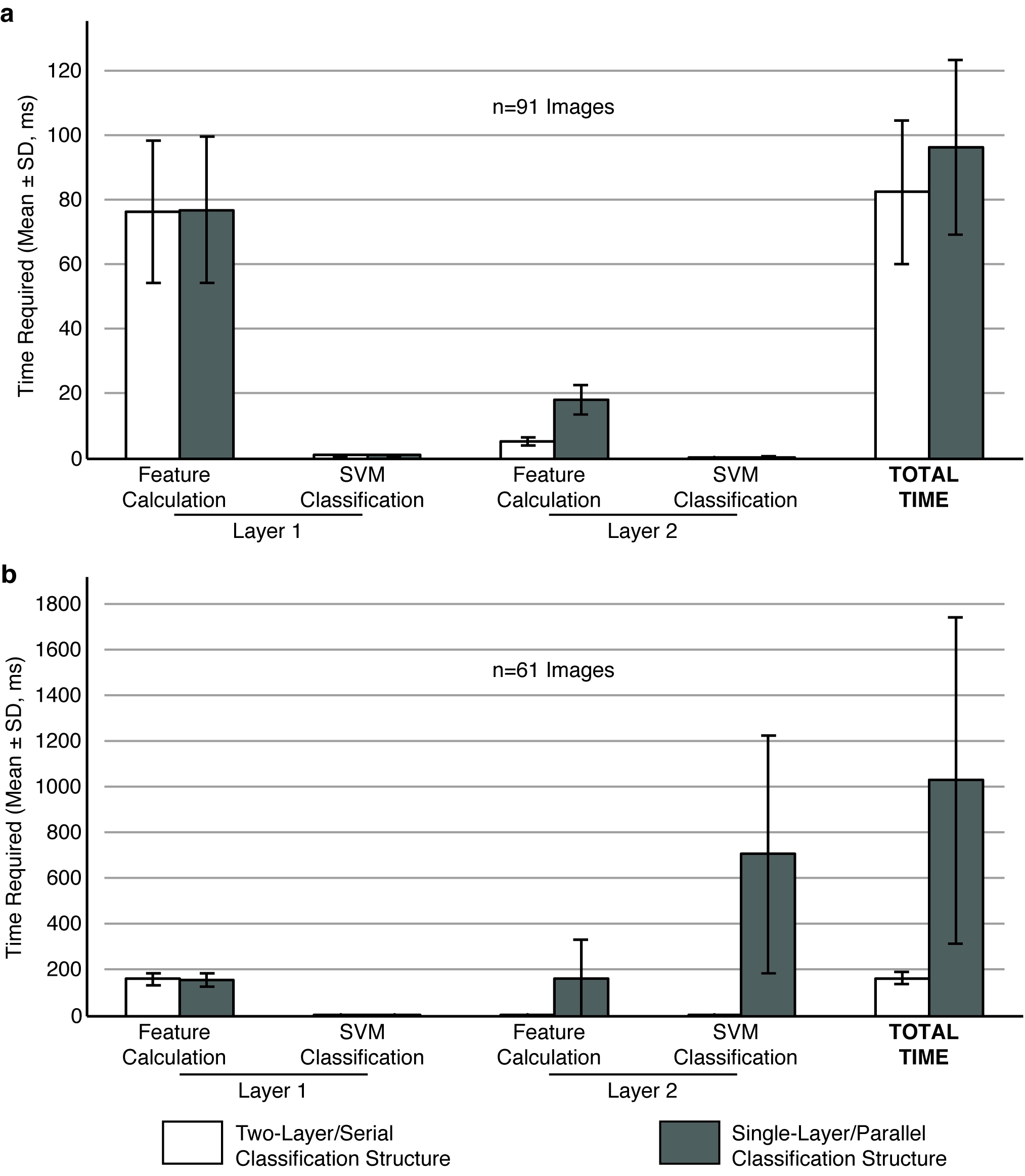

Supplement: S8 Fig — a) Comparison of process-specific and total time requirements for the two-layer and equivalent one-layer architectures when applied to single neuron pair detection. b) Comparison of process-specific and total time requirements for the two-layer and equivalent one-layer architectures when applied to the identification of two distinct neuron pairs. All times are based on performance on MATLAB 2013b running on a quad core processor at 3.50 GHz. (TIF) [file pcbi.1004194.s008.tif]

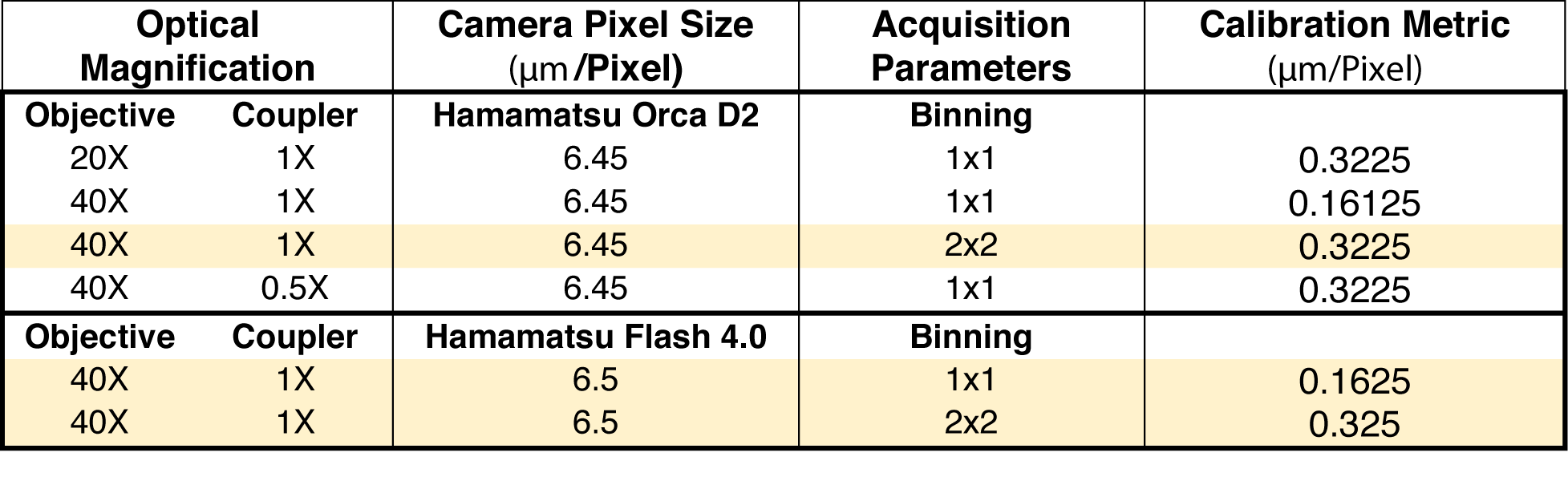

Supplement: S1 Table — Setups used for this study are highlighted. (TIF) [file pcbi.1004194.s009.tif]
